# Moroccan strains of *Leishmania major* and *Leishmania tropica* differentially impact on nitric oxide production by macrophages

**DOI:** 10.1186/s13071-017-2401-4

**Published:** 2017-10-23

**Authors:** Hasnaa Maksouri, Pham My-Chan Dang, Vasco Rodrigues, Jérôme Estaquier, Myriam Riyad, Khadija Akarid

**Affiliations:** 1 Center for Doctoral Studies on Health Sciences (Immunopathology), Faculty of Medicine and Pharmacy, Hassan II University of Casablanca (UH2C), Casablanca, Morocco; 2 Research team on Immunopathology of Infectious And Systemic Diseases, Laboratory of Cellular and Molecular Pathology, Faculty of Medicine and Pharmacy, UH2C, Casablanca, Morocco; 3 INSERM U1149/CRB3, Faculty of Medicine Xavier Bichat, Paris, France; 4 CNRS FR3636, Paris Descartes University, Paris, France; 5 Centre Hospitalier Universitaire (CHU) de Québec Research Center, Faculty of Medicine, Laval University, Québec, QC Canada; 6 Laboratory of Parasitology, Faculty of Medicine and Pharmacy, UH2C, Casablanca, Morocco; 7 Molecular Genetics and Immunophysiopathology research team, Health and Environment Laboratory, Aïn Chock Faculty of Sciences, UH2C, Casablanca, Morocco

**Keywords:** Cutaneous leishmaniasis, *Leishmania major*, *Leishmania tropica*, Soluble *Leishmania* antigens, macrophages, Nitric oxide, NO donors

## Abstract

**Background:**

Cutaneous leishmaniasis (CL) is a vector-borne parasitic disease caused by protozoa of the genus *Leishmania*. In Morocco, CL is a public health problem mainly caused by *Leishmania major* and *Leishmania tropica*, which are responsible for zoonotic and anthroponotic CL, respectively. Macrophages are the primary cells infected by *Leishmania* parasites and their capacity to produce nitric oxide (NO) is of critical importance for parasite elimination. To our knowledge, the role of NO on autochthonous infections has never been investigated before. In this study, we evaluated *in vitro* the capacity of autochthonous primary dermotropic strains of *L. major* and *L. tropica* to modulate NO production by J774-macrophages and determine the sensitivity of both species to exogenous NO.

**Methods:**

The infectivity of the J774 cell line was analyzed by optical microscopy. NO production by macrophages was measured by the Griess method. The sensitivity to NO by the two strains was assessed by the MTT assay using NO donors.

**Results:**

Our results show that the percentage of infected macrophages and the average number of parasites per macrophage were similar for *L. major* and *L. tropica* strains. While *L. tropica* significantly inhibited NO production induced by LPS and IFN-γ stimulation in J774 macrophages, *L. major* did not affect it. However, soluble *Leishmania* antigens (SLAs) from both autochthonous primary strains significantly inhibited the production of NO by J774-macrophages in a dose-dependent manner. Finally, our results demonstrated that promastigotes and amastigotes from both strains are sensitive to SNAP NO donor in a dose-dependent manner, although *L. tropica* demonstrated an increased sensitivity*.*

**Conclusions:**

Our results suggest a differential ability of *L. major* and *L. tropica* strains to modulate the capacity of macrophages to produce NO. The increased ability of *L. tropica* to inhibit NO production by macrophages might come as a necessity due to its higher sensitivity to NO donor. Our results provide one explanation for the tendency of *L. tropica* to cause chronic lesions and may contribute to the different physiopathology of CL in Morocco.

## Background

Leishmaniases are parasitic diseases caused by kinetoplastid protozoans of the family Trypanosomatidae, transmitted by the bite of infected female sand flies belonging to the genera *Phlebotomus* and *Lutzomyia* in the Old and New World, respectively [1]. Three main clinical forms of leishmaniasis are reported worldwide: cutaneous leishmaniasis (CL), visceral leishmaniasis (VL) and mucocutaneous leishmaniasis (MCL). They occur in 98 countries and, according to the World Health Organization (WHO), leishmaniasis affects 12 million people worldwide with approximately 0.2–0.4 million VL cases and 0.7–1.2 million CL cases diagnosed each year. In Morocco, CL is a major public health problem, with two main causative entities: the zoonotic form due to *Leishmania major* and the anthroponotic form due to *Leishmania tropica* [2]. Cutaneous lesions caused by these species are associated with a clinical polymorphism with respect to aspect, incubation period and healing time [3].

The parasite life-cycle involves two stages: the promastigote insect stage and the amastigote vertebrate stage. Promastigotes replicate and differentiate in the gut of hematophagous female sand fly vectors that inoculate metacyclic promastigotes into the mammalian host’s dermis when feeding. *Leishmania* parasites infect immune cells, particularly phagocytes, such as neutrophils, dendritic cells and particularly macrophages. Thus, the promastigotes develop into amastigotes, which multiply inside parasitophorous’ vacuoles in phagocytes [4].

*Leishmania* drastically alters the normal macrophage physiology, by modulating numerous signaling pathways [4]. For instance, we and others have reported that *Leishmania* species block apoptosis of their host cells, thus extending their viability [5, 6]. *In vitro* studies have also demonstrated that parasites modulate nitric oxide (NO) production, which exerts a leishmanicidal activity on promastigotes and amastigotes by inducing an apoptotic cell death program [7–9]. In murine systems, pro-inflammatory cytokines such as IFN-γ or TNFα, or endotoxins such as LPS, induce activation of iNOS, resulting in the elimination of intracellular parasites [10]. Mice deficient for iNOS are more sensitive to *L. major* or *L. donovani* infection. Moreover, the C57BL/6 mice resistance correlates with the ability of macrophages to produce NO following activation. In contrast, BALB/c mice are susceptible to *L. major* infection due to the lower level of iNOS activation and NO production by macrophages [7, 11]. Furthermore, it has been shown that *L. amazonensis*-infection of C57BL/6 mice is controlled via NO and reactive oxygen species (ROS) produced by macrophages [12]. In patients infected with *L. tropica*, biopsies and serosities sampled at early stages of the infection showed early iNOS expression and presence of NO in the sera; similar observations are reported in patients infected by *L. donovani* [13, 14]. In contrast, other species have developed a resistance to NO produced by activated macrophages such as *L. chagasi* promastigotes [15, 16]. Because the maintenance of *Leishmania* spp. *in vitro* results in a progressive loss of virulence [17], it is of crucial importance to use primary isolates for assessing CL pathogenicity.

Thus, our aim was to determine whether the pathogenicity of clinical *L. major* or *L. tropica* primary isolates from Moroccan patients is related to a difference in (i) their capacity to modulate the NO production by macrophages, and/or (ii) their susceptibility to exogenous NO.

## Methods

### *Leishmania* strains

*Leishmania major* (MHOM/MA/2010/L112) and *L. tropica* (MHOM/MA/2010/L02) strains were isolated from skin lesions of Moroccan CL patients diagnosed in the Department of Dermatology (Ibn Rochd Hospital of Casablanca, Morocco). The dermal syringe-sucked fluid was collected under sterile conditions from the border of active skin lesions from each patient as follows: the lesions were cleaned with alcohol, and 0.1 to 0.2 ml of sterile saline solution was injected using a 1 ml syringe (25-gauge needle) into the nodule and the needle was rotated gently several times. A small amount of saline solution was injected into the tissue, and then aspirated. They were subsequently directly genotyped in the Parasitology Laboratory at the Casablanca Faculty of Medicine and Pharmacy according to Mouttaki et al. [18]. The promastigotes were isolated in NNN biphasic medium and then grown and maintained at 26 °C in RPMI-1640 medium (Gibco, Essonne, France) supplemented with 10% heat-inactivated fetal calf serum (Gibco), 2 mM L-glutamine (Gibco), 100 U/ml penicillin (Gibco), and 100 ng/ml streptomycin (Gibco). *Leishmania major* and *L. tropica* promastigotes were used after 5 successive in vitro passages.

### Soluble *Leishmania* promastigotes antigens (SLAs)

SLAs were prepared from stationary phase promastigotes of *L. major* and *L. tropica* strains as described by [19]. Briefly, the promastigotes were washed 3 times in cold sterile phosphate-buffered saline (PBS) and then adjusted to 10^8^ promastigotes/ml in PBS. The parasites were disrupted by ten cycles of freezing (-80 °C) and thawing (37 °C) followed by ultra-sonication (20 times for 10 s). SLAs were aliquoted and stored at -80 °C until use. The protein concentration was determined by Bradford protein assay (Protein Assay kit, BioRad, New York, USA).

### *In vitro* infection of the murine J774 cell line by *Leishmania* promastigotes

The murine macrophage J774 cell line, cultured at 37 °C at 5% CO_2_, was seeded at the density of 5.10^5^ cells/well in 24-well culture plates on round 12 mm diameter cover-slips (Greiner Bio-One International, Frickenhausen, Germany). After overnight culture, promastigotes were added at ratio 10 and 5 parasites per macrophage (ratio 10:1 and 5:1) and incubated at 37 °C for 4 h. Infected cells were washed 3 times with sterile PBS to remove extracellular promastigotes. The coverslips were removed from the plates and stained with Giemsa dye 24 and 48 h post-infection. Infected cells and intracellular parasites were counted in ten fields under a light microscope by two independent observers. The rate of infected macrophages was calculated as follows: (Number of infected macrophages/Total number of macrophages) × 100. The amastigote infection rates were estimated by the mean number of intracellular amastigotes in 100 cells.

### Promastigotes viability control in LPS/IFN-γ stimulated macrophages

The murine macrophage J774 cell line was cultured as described above. After 48 h post-infection the supernatant was removed and cells were washed and then incubated with 0.01% SDS for 10 min to allow release of live parasites. Then 1 ml of RPMI-1640 medium supplemented with 10% FCS was added to each well, and the plate was incubated at 26 °C for 3 days. The relative intracellular load of parasites was measured by assessing the number of extracellular motile promastigotes produced in medium using the Trypan blue exclusion assay.

### Nitric oxide (NO) production

The cells were stimulated in the absence or presence of either IFN-γ (10 ng/ml) (Sigma-Aldrich, Taufkirchen, Germany) and Lipopolysaccharide (LPS) (50 ng/ml) (Sigma-Aldrich) in presence or absence of N^G^-Methyl-L-arginine acetate salt (L-NMMA) (1 mg/ml) (Sigma-Aldrich). The cells were also stimulated with SLAs (10 μg/ml or 20 μg/ml). Supernatants were collected 24 and 48 h post-stimulation and stored at -20 °C until use for nitrite assay.

Nitrite (NO_2_^−^) is a stable compound produced by the reaction of NO with water and oxygen, and its accumulation reflects the amount of NO produced in culture medium. NO_2_^−^ assay was performed according to Griess protocol previously described [20]. Briefly, 100 μl of supernatants were distributed to 96-well plates in triplicates and 100 μl of Griess reagent (1% sulfanilamide, 0.1% N-(1-Naphthyl) ethylenediamine dihydrochloride and 2.5% H_3_PO_4_) (Sigma-Aldrich) was added. The plates were incubated at room temperature and optical density (OD) was read at 540 nm using a BioRad Micro-plate Reader Photometer (BioRad Dynex, Washington, USA). The NO concentration was calculated from a standard curve generated with NaNO_2_ ranging from 0 to 200 μM. The experiments were performed in triplicates and the results represent the average of 6 independent experiments.

### Impact of exogenous NO on parasite viability

#### Promastigote viability

Promastigotes at stationary phase were incubated at 5.10^5^ parasites/well in 96-well plates in the presence of different concentrations of S-Nitroso-N-acetylpenicillamine (SNAP) (Sigma-Aldrich), and N-acetyl-D-L-penicillamine (NAP) (Sigma-Aldrich) ranging from 25 to 100 μM. The plates were incubated at 26 °C for 24 h and the viability rate was determined by MTT assay. Briefly, 10 μl of MTT reagent (5 mg/ml) [3-(4, 5-dimethythiazol-2-yl)-2, 5-diphenyltetiazolium bromide] (Sigma-Aldrich) was added and incubated at 26 °C for 4 h. Solubilization of the formed formazan crystals was performed by using 100 μl of acidic isopropanol 0.04 N and 50 μl of DMSO. After incubation for 15 min at room temperature; the OD was measured at 570 nm using a BioRad Micro-plate Reader Photometer (BioRad Dynex, Washington, USA). The promastigote viability index was assessed as following: (absorbance of treated promastigotes/absorbance of control promastigotes) × 100. The 50% inhibitory concentration (IC_50_), i.e. the SNAP concentration that decreases the growth by 50%, was calculated by regression analysis [21]. Results represent the average of 6 independent experiments.

#### Amastigote viability

Cell monolayers of infected macrophages treated or untreated with SNAP for 24 h were washed and incubated with 0.01% SDS for 10 min to allow release of live amastigotes. 1 ml of RPMI-1640 medium supplemented with 10% FCS was added to each well, and the plate was incubated at 26 °C for 2 days. The relative intracellular load of amastigotes was measured by assessing the number of extracellular motile promastigotes produced in the medium [22].

### Statistical analysis

Data are represented as mean ± SEM. The significance of the results was calculated using one-way ANOVA analysis of variance with Tukey’s *post-hoc* test for multiple comparisons was implemented using GraphPad Prism version 5.0. The threshold of significance was fixed at *P* < 0.05.

## Results

The clinical phenotypes of patients from which the two primary strains of *L. major* (MHOM/MA/2010/L112) and *L. tropica* (MHOM/MA/2010/L02) were isolated are summarized in Table 1. Both strains were isolated from patients living in Casablanca, a non-endemic area for CL. They were both infected during a summer staying in known endemic foci of CL due to *L. tropica* or *L. major*.

**Table 1:** Clinical profile of patients from which the two strains were isolated

Strains	MHOM/MA/2010/L02	MHOM/MA/2010/L112
Patients	Patient 1	Patient 2
Age (years)	5	21
Sex	Female	Male
Residence city	Casablanca	Casablanca
Main clinical data:		
Number of dermal lesions	1	5
Incubation time (months)	1	1–3^a^
Evolution time of lesions at diagnosis (months)	4	2
Evolution of lesions upon treatment	Favorable but needed more than a single cure	Favorable
Genotyping	*L. tropica*	*L. major*

^a^The exact incubation time could not be determined because the patient reported two distinct staying periods in endemic foci

### Infection of the J774 cell line by *L. major* and *L. tropica* primary strains

**Fig. 1:**
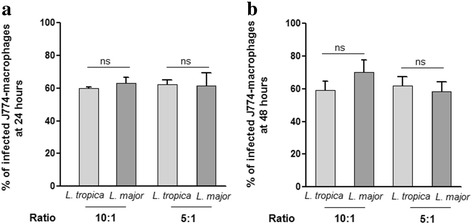
Percentage of *J774* macrophage-like cells infected by *L. major* and *L. tropica* strains. After 24 h (**a**) and 48 h (**b**) of culture, the percentage of infected cells per 100 cells was quantified microscopically. Results are expressed as mean ± SEM from three independent experiments, each performed in duplicate. Data were analyzed using one way ANOVA test with Tukey’s *post-hoc* test for multiple comparisons. *Abbreviation*: ns, not significant

To compare the infectivity of both *L. major* and *L. tropica* primary isolates, we employed the well-established J774 cell line as a model for *Leishmania* infection. The percentage of macrophages infected by *L. major* and *L. tropica* strains was 60 and 68%, respectively. No significant difference was observed when comparing the percentage of infected cells at 24 h post-infection (Fig. 1a) and 48 h post-infection (Fig. 1b). Furthermore, we did not observe any major difference at either a 10:1 or 5:1 parasite-to-host cell ratios. For both infection ratios, we found an average 3–4 amastigotes/cell (Table 2) and these values were similar for both strains. Thus our results show no difference in the infectivity of both *L. major* and *L. tropica* strains*.*

**Table 2:** Amastigote infection rates of J774-macrophages (mean ± SEM)^a^

	*L. tropica* strain		*L. major* strain	
Ratio	10:1	5:1	10:1	5:1
Incubation time				
24 h	4.0 ± 0.3	3.0 ± 0.3	3.0 ± 0.3	3.0 ± 0.3
48 h	3.0 ± 0.2	3.0 ± 0.3	4.0 ± 0.3	3.5 ± 0.5

^a^Results are expressed as mean ± SEM from three independent experiments

### Modulation of NO production by *L. major* and *L. tropica* promastigotes

**Fig. 2:**
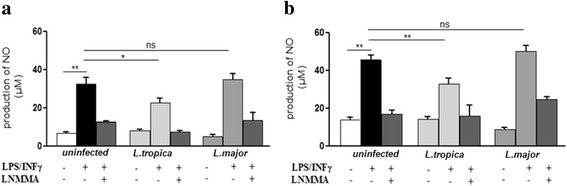
Effect of *L. major* and *L. tropica* promastigotes on NO production by macrophages. Macrophages were infected with 10 promastigotes of *L. tropica* or *L. major* /cell for 4 h. Cells were kept either without stimulus or stimulated with IFN-γ (10 ng/ml) and LPS (50 ng/ml) in presence or absence of L-NMMA (1 mg/ml). The supernatants were collected and nitrite concentrations were evaluated by Griess reaction at 24 h post-infection (**a**) and 48 h post-infection (**b**). Results are expressed as mean ± SEM from six independent experiments, each performed in duplicate. Data were analyzed using one way ANOVA test with Tukey’s *post-hoc* test for multiple comparisons. *P < 0.05, **P < 0.01. *Abbreviation*: ns, not significant

We next wanted to assess whether the two primary *L. major* and *L. tropica* clinical isolates strains modulate the NO production by macrophages. Macrophages were infected at two ratios (10:1 and 5:1), and stimulated with IFN-γ and LPS. Nitrite concentrations in supernatants were measured at 24 and 48 h post-infection. In the absence of stimulation, none of the strains induced NO production (Fig. 2a, b). Stimulated uninfected macrophages produced levels of NO ranging from 29 to 42 μM at 24 h (*F*_(3,20)_ = 30.81, *P =* 0.003) and 48 h (*F*_(3,20)_ = 42.36, *P =* 0.002), respectively. In the presence of L-NMMA, an iNOS inhibitor, NO production was suppressed, thus showing the specificity of the measurement. The results obtained with 5:1 ratio did not differ from 10:1 results. So, we presented only 10:1 ratio results.

Our results showed that *L. tropica* infection significantly reduced the production of NO induced by IFN-γ and LPS at 24 h (*F*_(2,15)_ = 7.01, *P =* 0.04) and 48 h (*F*_(2,15)_ = 9.33, *P =* 0.002), compared to stimulated non-infected cells. In contrast, infection with *L. major* had no impact on NO production after stimulation (Fig. 2a, b). Therefore, our results show that primary autochthonous *L. major* and *L. tropica* strains differentially modulate IFN-γ and LPS-induced NO production by macrophages*.*

### Promastigotes viability control in LPS/IFN-γ stimulated macrophages

**Fig. 3:**
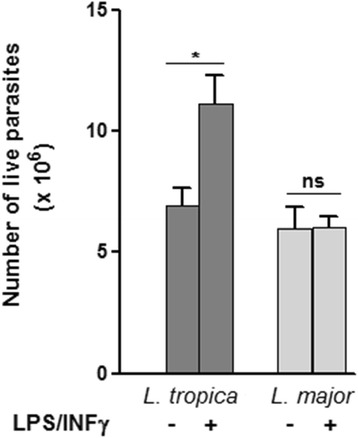
Number of *L. tropica* and *L. major* promastigotes in LPS/IFN-γ activated macrophages. Number of extracellular motile promastigotes was counted after 72 h incubation using the Trypan blue exclusion assay. Results are presented as mean ± SEM from three independent experiments, each performed in duplicate. Data were analyzed using one way ANOVA test with Tukey’s *post-hoc* test for multiple comparisons. *P < 0.05. *Abbreviation*: ns: not significant

The *L. tropica* and *L. major* viability control was investigated in macrophages after stimulation. As shown in Fig. 3, the number of viable *L. tropica* promastigotes after stimulation was significantly increased compared to in unstimulated macrophages (*F*_(3,20)_ = 8.21, *P =* 0.035). Thus, the high NO inhibition by *L. tropica* promastigotes shown in Fig. 2 is associated with a significant parasite load. However, in *L. major*-infected macrophages, there was no difference in parasite number between stimulated and unstimulated cells.

### Modulation of NO production by *L. major* and *L. tropica* SLAs

**Fig. 4:**
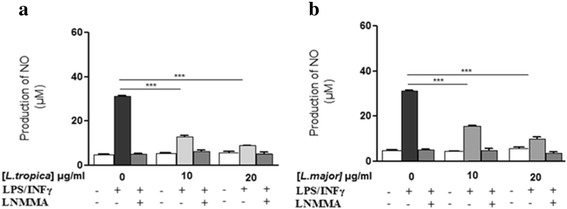
Effect of *L. major* and *L. tropica* SLA on NO production by macrophages. The J774 macrophages cells were cultured in presence of *L. tropica* SLA (**a**), *L. major* SLA (**b**), and stimulated according to the same protocol than promastigotes. The supernatants were collected and nitrite concentrations were evaluated by Griess reaction after 24 h post-infection. Results are expressed as mean ± SEM from three independent experiments, each performed in duplicate. Data were analyzed using one way ANOVA test with Tukey’s post-hoc test for multiple comparisons. ***P < 0.001

Several components derived from parasites, such as the surface glycolipid lipophosphoglycan (LPG) and the metalloprotease GP63 which impact on phagosome and function of the NADPH oxidase complex, modulate intracellular signaling [23]. Furthermore, the protein tyrosine phosphatase SHP-1 (Src-homology 2 domain containing phosphatase-1) has been reported to inhibit NO production [24]. Therefore, we decided to determine whether soluble *Leishmania* antigens (SLAs), derived from either *L. major* or *L. tropica* strains, modulate NO production. Thus, SLAs from *L. tropica* and *L. major* significantly inhibited the production of NO by J774 (Fig. 4a, b). However, unlike infection with live parasites, no difference was observed between the two strains. These results suggest that distinct parasite factors and distinct mechanisms are responsible for inhibition of NO production by the two strains studied.

### *Leishmania major* and *L. tropica* susceptibility to exogenous NO

NO is highly toxic to *Leishmania* parasites, leading to mitochondrial permeabilization and dysfunction. We employed the MTT assay to measure parasite viability in the presence of increasing concentrations of SNAP, a NO donor, compared to a control molecule, NAP. Given that the conversion of MTT to formazan is performed mostly by mitochondrial reductases, the assay also serves as a marker of mitochondrial activity.

**Fig. 5:**
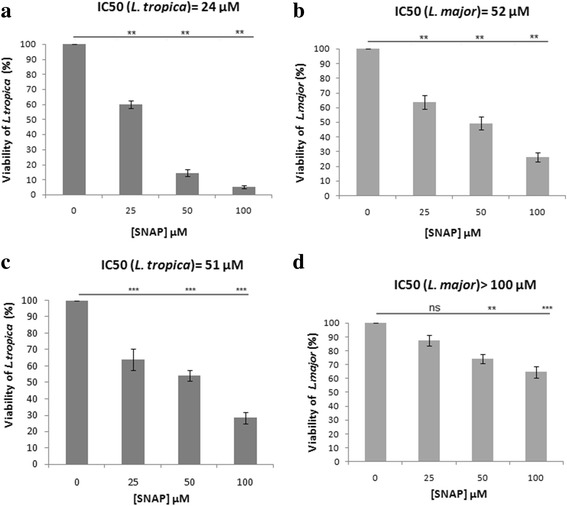
*Leishmania major* and *L. tropica* promastigotes and amastigotes sensitivity to exogenous NO. Viability of *L. tropica* promastigotes (**a**) and viability of *L. major* promastigotes (**b**) and amastigotes (**c**, **d**) was evaluated after 24 h incubation with different concentrations of SNAP and its control (NAP). In all experiments, the addition of NAP at the same concentrations as SNAP did not impact the viability of parasites (100% viability). Results are expressed as mean ± SEM from six independent experiments, each performed in duplicate. The NO release by SNAP was measured in the culture supernatant for each concentration. NO concentration ranges: **a** and **b**: 17.26–55.20 μM, **c** and **d**: 14.75–44.67 μM. Data were analyzed using one way ANOVA test with Tukey’s *post-hoc* test for multiple comparisons.**P < 0.01, ***P < 0.001. *Abbreviation*: ns: not significant

Our results showed that SNAP significantly reduces the viability of promastigotes of both *L. tropica* and *L. major* strains, in a dose-dependent manner. Indeed at 25 μM (*F*_(3,20)_ = 800.6, *P* = 0.002), 50 μM (*F*_(3,20)_ = 800.6, *P* = 0.005) and 100 μM (*F*_(3,20)_ = 800.6, *P* = 0.0019), the viability of *L. tropica* (Fig. 5a) and *L. major* (Fig. 5b) promastigotes was significantly reduced compared to control promastigotes (*F*_(3,20)_ = 144, *P* = 0.0039). After 24 h of incubation, the viability decrease of *L. tropica* promastigotes was more important at the dose of 50 μM and 100 μM of SNAP. The IC_50_ of SNAP was 24 μM and 52 μM for *L. tropica* and *L.major* promastigotes, respectively (Fig. 5a, b).

We then assessed the impact of SNAP on amastigote forms, which are the intracellular forms in infected host-cell targets. Our results demonstrated that both are significantly sensitive in a dose-dependent manner. The viability of *L. tropica* amastigotes decreased significantly at 25 μM, 50 μM and 100 μM (*F*_(3,8)_ = 55, *P* = 0.0005). The viability of *L. major* amastigotes decreased significantly only at 50 μM (*F*_(3,8)_ = 21.18, *P* = 0.002) and 100 μM (*F*_(3,8)_ = 21.18, *P* = 0.0004). The viability decrease of *L. tropica* amastigotes was more significant than for *L. major* (Fig. 5c, d). The SNAP concentrations 25 μM and 50 μM did not have a significant effect on the viability of uninfected J774 macrophages (data not shown). The control molecule (NAP) had no effect on the viability of promastigotes and amastigotes of both strains (100% viability).

In order to verify NO release by SNAP, we measured the nitrite accumulation in the cultures supernatants at 24 h of incubation. These concentrations were correlated with the input amount of SNAP concentrations. Indeed, NO concentrations ranges were 17.26–55.20 μM for promastigotes and 14.75–44.67 μM for amastigotes. Thus, our results demonstrate that both promastigotes and amastigotes of *L. tropica* display higher sensitivity to exogenous NO compared to *L. major.*

## Discussion

Cutaneous leishmaniasis remains a major public health problem in Morocco with thousands of new cases occurring each year. This number reached 8707 patients diagnosed in 2010, and owing to the control measures implemented by the Ministry of Health, this number decreased to 2555 in 2014 [25]. Clinical manifestations of the infection depend upon the infecting *Leishmania* species and the efficacy of the host immune response to control and eliminate the parasite. The co-evolutionary process enabled *Leishmania* parasites to adapt and survive in the myeloid cell lineage [26].

Herein, we demonstrate a differential ability to modulate macrophages NO production by two primary autochthonous *L. tropica* and *L. major* strains isolated from Moroccan patients. While both strains exhibit similar J774 macrophages-infectivity, the *L. tropica* strain has an increased capacity to inhibit NO production as compared with *L. major* strain. Our results further show that both *L. tropica* promastigotes and amastigotes are more sensitive to NO donor, than *L major* promastigotes and amastigotes. Conversely, *in vitro* studies have shown that the SNAP or S-nitrosoglutathione (GSNO) NO donors have a leishmanicidal activity on promastigotes and amastigotes of *L. major* or *L. amazonensis* [27, 28]. The local treatment of skin lesions of patients suffering from CL due to *L. major* with a SNAP-containing cream accelerates the healing process. Similarly, the GSNO was also used efficiently to locally treat ulcerated skin lesions in mice infected with *L. major* [29]. Susceptible BALB/c mice infected with *L. major* and treated with NO donors (GSNO or trinitroglycerin) show a reduction in parasite burden at the infection site and a marked reduction in disease progression.

Although expression of NO might be a critical leishmanicidal marker, it has been reported that some parasite strains escape to this process. Human and canine *L. infantum* and *L. braziliensis* strains are resistant to NO released by exogenous donor, which is associated with the clinical worsening of human and canine leishmaniasis [15, 28]. Moreover, it has been shown that despite a strong expression of NO in the serum of CL patients due to *L. tropica* [13, 14] or iNOS in the spleen of *L. chagasi-*infected dogs, these infections are associated with a parasite burden increase [30]. In murine macrophages infected either by *L. infantum* or *L. chagasi,* a high NO production was also associated with a significant parasite load, indicating that both species are able to survive and resist to NO produced in macrophages. Hence some *Leishmania* species are sensitive to NO while other species would be able to develop resistance to endogenous NO leading to a worsening course of the disease [30, 31].

Herein, we demonstrated that SNAP significantly reduces the viability of promastigote and amastigote forms of both strains in a dose-dependent manner. However, the *L tropica* strain was more sensitive than *L major* strain. It has been proposed that the clinical diversity of CL dermal lesions results not only from the different parasite species involved or the type of the zoonotic cycle concerned but also the immunological status and the genetic background of the patients [32, 33]. *Leishmania tropica* clinical strains were isolated from a 5 years old girl living in Casablanca that spent her holidays in a known endemic focus of CL. One cutaneous lesion appeared 1 month later and she consulted 4 months later. The delayed healing process of the lesion led to several cures. Healing of lesions due to *L. tropica* may last more than 1 year, confirming the chronic tendency of this form of CL. Relapses and treatment failures are also not exceptional for this pathology [1, 34]. The second patient presented 5 dermal lesions that healed correctly under the standard treatment. The classical lesion caused by *L. major* is of “wet” type and heals within 2–8 months [34, 35]. Although we cannot exclude that host factors, such as age, may have contributed to a long healing process that needed more than one cure, it may also be due to a more virulent *L. tropica* strain. Furthermore we showed the increased ability of this *L. tropica* strain to alter NO production. If our ongoing assays on other Moroccan *L. tropica* strains confirm this tendency, we could consider that this intrinsic character may foster the geographical spread of *L. tropica* throughout Morocco reported since the 1990s; *L. tropica* foci were limited to central south regions in the 1980s [1].

Our results indicate that the reduction of NO production by *L. tropica* infected and stimulated macrophages is associated with a significant increase of motile promastigotes produced by these cells. This may suggest that the intracellular macrophage control of the *L. tropica* strain do not only rely on NO. Other key molecules, like ROS, are involved in the macrophage-mediated innate host defense against *Leishmania*. Despite several studies on ROS and NO species in *Leishmania* killing, their mechanism is still misunderstood. On the other hand, it has been reported that some inflammatory cytokines produced by infected phagocytes shifts the L-arginine metabolism towards the production of L-ornithine and urea through arginase activation, leading to a decrease in NO secretion that fosters intracellular *Leishmania* growth. Furthermore it was reported that IFN-γ can act as a growth factor within macrophages for some *Leishmania* species (i.e. *L. amazonensis*). Thus the subversion of NO production by *Leishmania* is well recognized as a mechanism of immune escape, allowing the parasite to survive for a longer time [7].

A large number of studies have implicated the importance of glycoconjugates in New or Old World *Leishmania* biology. These glycoconjugates include LPG, glycoinositolphospholipids (GIPLs), gp63 and the proteophosphoglycan (PPG). It is well documented that glycoconjugate interspecies polymorphism is important for the differential regulation of initial events of the immune response, as well as in the establishment of the infection [36]. LPG from *L. major* induced the production of IFN-γ and the expression of iNOS, resulting in host protection and parasite resistance in the mouse model [7, 37]. *Leishmania infantum* LPG also triggered higher NO production [36, 38]. Interestingly, macrophages stimulated with *L. braziliensis* LPG had a higher TNFα, IL-1α, IL-6 and NO production than those stimulated with *L. infantum*, while a strong inhibition of NO production by LPS was observed after LPG incubation from both species [38]. LPG from *L. tropica* is the most complex characterized to date, as most of the repeat units are substituted with more than 19 different glycan side chains [37]. Furthermore, Ghoshal et al. [39] showed that *Leishmania* species have a differential distribution of sialic acid (SA) on their surface despite their close pathogenesis resemblance. Thus, enhanced higher levels of SA serve as one of the potential determinants responsible for increased NO resistance and survivability of parasites by inhibition of host responses [39]. In the present study, using SLA derived from *L. major* and *L. tropica* strains, we demonstrated a diminution of NO production in IFN-γ/ LPS stimulated macrophages.

This result is of importance given that it indicates that live pathogens differ in their ability to modulate NO production compared to that observed with SLAs. We have previously reported that only live parasites were capable to modulate macrophage’s death [5]. Furthermore, we recently demonstrated that infected macrophages, and not only those exposed to parasites (bystander), modulate cell host bioenergetics profile [40], and affect dendritic function in inducing Th1 CD4 T cells expressing IL-10 [41].

These first experiments were carried out with primary dermal strains passively isolated from patients consulting at Ibn Rochd Hospital of Casablanca (Morocco), which is not an endemic area of CL. The results showed a different NO modulation by these two strains representing *L. major* and *L. tropica* species*.* This led us to plan further experiments with additional strains from different endemic regions.

## Conclusions

In Morocco, as well as in the other North African countries, CL due to *L. major* and *L. tropica* remains a serious public health concern. Our results suggest a distinct capacity of our primary strains to modulate NO production in order to survive in infected macrophages, which in turn may explain the different physiopathology of these species, especially the chronic tendency of *L. tropica* CL. *Leishmania tropica* is recognized as a very heterogeneous species and its intraspecific heterogeneity is readily demonstrated by many authors. Human infection caused by *L. tropica* seems to be more insidious compared with *L. major* infection. Therefore, although it remains to extend this observation to additional clinical primary strains, understanding the molecular mechanisms by which *L. tropica* subverses NO production could be essential for the control of this endemic infection.

## Abbreviations

CL: Cutaneous leishmaniasis
iNOS: Inducible nitric oxide synthase
L-NMMA: N^G^-Methyl-L-arginine acetate salt
LPS: Lipopolysaccharide
MTT: 3-(4, 5-dimethythiazol-2-yl)-2, 5-diphenyltetiazolium bromide
NAP: N-acetyl-D-L-penicillamine
NO: Nitric oxide
SLAs: Soluble *Leishmania* antigens
SNAP: S-Nitroso-N-acetylpenicillamine

## References

[1] Aoun K, Bouratbine A (2014). Cutaneous leishmaniasis in North Africa: a review. Parasite.

[2] Rhajaoui M. [Human leishmaniases in Morocco: a nosogeographical diversity.] Pathologie-biologie. 2011;59:226–9. (in French). 10.1016/j.patbio.2009.09.003 19942371

[3] Er-Rami M, Benjelloun S, Lahlou H, Khalloufi A, El Kartouti A, Zeroual A, et al. [Cutaneous leishmaniasis in the military hospital Moulay Ismail of Meknes (Morocco): about 49 cases diagnosed between 2005 and 2011.] Pathologie-biologie. 2013;61:49–53. (in French). 10.1016/j.patbio.2012.03.009 22542427

[4] Duque GA, Descoteaux A (2015). *Leishmania* survival in the macrophage: where the ends justify the means. Curr Opin Microbiol.

[5] Akarid K, Arnoult D, Micic-Polianski J, Sif J, Estaquier J, Ameisen JC (2004). *Leishmania major*-mediated prevention of programmed cell death induction in infected macrophages is associated with the repression of mitochondrial release of cytochrome c. J Leukoc Biol.

[6] Rodrigues V, Cordeiro-da-Silva A, Laforge M, Ouaissi A, Silvestre R, Estaquier J (2012). Modulation of mammalian apoptotic pathways by intracellular protozoan parasites. Cell Microbiol.

[7] Horta MF, Mendes BP, Roma EH, Noronha FSM, Macêdo JP, Oliveira LS, et al. Reactive oxygen species and nitric oxide in cutaneous leishmaniasis. J Parasitol Res. 2012;203818. 10.1155/2012/203818 PMC3337613 22570765

[8] Das M, Mukherjee SB, Shaha C (2001). Hydrogen peroxide induces apoptosis-like death in *Leishmania donovani* promastigotes. J Cell Sci.

[9] Arnoult D, Akarid K, Grodet A, Petit P, Estaquier J, Ameisen JC (2002). On the evolution of programmed cell death: apoptosis of the unicellular eukaryote *Leishmania major* involves cysteine proteinase activation and mitochondrion permeabilization. Cell Death Differ.

[10] Kuang Z, Lewis RS, Curtis JM, Zhan Y, Saunders BM, Babon JJ (2010). The SPRY domain-containing SOCS box protein SPSB2 targets iNOS for proteasomal degradation. J Cell Biol..

[11] Liew FY, Li Y, Moss D, Parkinson C, Rogers MV, Moncada S (1991). Resistance to *Leishmania major* infection correlates with the induction of nitric oxide synthase in murine macrophages. Eur J Immunol.

[12] Côrtes DF, Carneiro MBH, Santos LM, TCdO S, Maioli TU, Duz AL (2010). Low and high-dose intradermal infection with *Leishmania major* and *Leishmania amazonensis* in C57BL/6 mice. Mem Inst Oswaldo Cruz.

[13] Kumar R, Bumb RA, Salotra P (2010). Evaluation of localized and systemic immune responses in cutaneous leishmaniasis caused by *Leishmania tropica*: interleukin-8, monocyte chemotactic protein-1 and nitric oxide are major regulatory factors. Immunology.

[14] Serarslan G, Atik E (2005). Expression of inducible nitric oxide synthase in human cutaneous leishmaniasis. Mol Cell Biochem.

[15] Santos P, Costa R, Braz J, Santos L, Batista A, Vasconcelos CR (2012). *Leishmania chagasi* naturally resistant to nitric oxide isolated from humans and dogs with visceral leishmaniasis in Brazil. Nitric Oxide.

[16] Gantt KR, Schultz-Cherry S, Rodriguez N, Jeronimo SM, Nascimento ET, Goldman TL (2003). Activation of TGF-β by *Leishmania chagasi*: importance for parasite survival in macrophages. J Immunol.

[17] Moreira D, Santarém N, Loureiro I, Tavares J, Silva AM, Amorim AM (2012). Impact of continuous axenic cultivation in *Leishmania infantum* virulence. PLoS Negl Trop Dis.

[18] Mouttaki T, Morales-Yuste M, Merino-Espinosa G, Chiheb S, Fellah H, Martin-Sanchez J, Riyad M (2014). Molecular diagnosis of cutaneous leishmaniasis and identification of the causative *Leishmania* species in Morocco by using three PCR-based assays. Parasit Vectors.

[19] Dey R, Majumder N, Majumdar SB, Bhattacharjee S, Banerjee S, Roy S, Majumdar S. Induction of host protective Th1 immune response by chemokines in *Leishmania donovani*-infected BALB/c mice. Scand J Immunol. 2007;66:671–83. 10.1111/j.1365-3083.2007.02025.x 18021365

[20] Akarid K, Sinet M, Desforges B, Gougerot-Pocidalo MA (1995). Inhibitory effect of nitric oxide on the replication of a murine retrovirus in vitro and in vivo. J Virol.

[21] Rudrapaul P, Sarma IS, Das N, De UC, Bhattacharjee S, Dinda B. New flavonol methyl ether from the leaves of *Vitex peduncularis* exhibits potential inhibitory activity against *Leishmania donovani* through activation of iNOS expression. Eur J Med Chem. 2014;87:328–35. 10.1016/j.ejmech.2014.09.076 25264585

[22] Ribeiro-Gomes FL, Moniz-de-Souza MCA, Alexandre-Moreira MS, Dias WB, Lopes MF, Nunes MP (2007). Neutrophils activate macrophages for intracellular killing of *Leishmania major* through recruitment of TLR4 by neutrophil elastase. J Immunol.

[23] Lodge R, Diallo TO, Descoteaux A (2006). *Leishmania donovani* lipophosphoglycan blocks NADPH oxidase assembly at the phagosome membrane. Cell Microbiol.

[24] Forget G, Gregory DJ, Whitcombe LA, Olivier M (2006). Role of host protein tyrosine phosphatase SHP-1 in *Leishmania donovani*-induced inhibition of nitric oxide production. Infect Immun.

[25] Ministry of Health: Santé en chiffres 2014. Direction de la Planification et des Ressources Financières; Edition 2015. http://www.sante.gov.ma/Publications/Etudes_enquete/Pages/default.aspx.

[26] Schmid M, Zimara N, Wege AK, Ritter U (2014). Myeloid-derived suppressor cell functionality and interaction with *Leishmania major* parasites differ in C57BL/6 and BALB/c mice. Eur J Immunol.

[27] Costa ISF, de Souza GFP, de Oliveira MG, de Almeida Abrahamsohn I (2013). S-nitrosoglutathione (GSNO) is cytotoxic to intracellular amastigotes and promotes healing of topically treated *Leishmania major* or *Leishmania braziliensis* skin lesions. J Antimicrob Chemother.

[28] Van Assche T, Deschacht M, da Luz RAI, Maes L, Cos P. *Leishmania-*macrophage interactions: Insights into the redox biology. Free Rad Biol Med. 2011;51:337–51. 10.1016/j.freeradbiomed.2011.05.011 21620959

[29] de Souza GFP, Yokoyama-Yasunaka JK, Seabra AB, Miguel DC, de Oliveira MG, Uliana SR (2006). Leishmanicidal activity of primary S-nitrosothiols against *Leishmania major* and *Leishmania amazonensis*: implications for the treatment of cutaneous leishmaniasis. Nitric Oxide.

[30] Gantt KR, Goldman TL, McCormick ML, Miller MA, Jeronimo SM, Nascimento ET (2001). Oxidative responses of human and murine macrophages during phagocytosis of *Leishmania chagasi*. J Immunol.

[31] dos Santos FR, Vieira PMA, Correa-Oliveira R, Giunchetti RC, Carneiro CM, Reis AB, Malaquias LC (2011). Qualitative and quantitative immunohistochemical evaluation of iNOS expression in the spleen of dogs naturally infected with *Leishmania chagasi*. Parasitol Res.

[32] Reithinger R, Dujardin J-C, Louzir H, Pirmez C, Alexander B, Boooker S (2007). Cutaneous leishmaniasis. Lancet Infect Dis.

[33] World Health Organization: Control of the leishmaniases. Report of a meeting of the WHO experts committee on the control of leishmaniases, Geneva; 22–26 March 2010. http://apps.who.int/iris/bitstream/10665/44412/1/WHO_TRS_949_eng.pdf

[34] Chiheb S, Guessous-Idrissi N, Hamdani A, Riyad M, Bichichi M, Hamdani S, Krimech A. *Leishmania tropica* cutaneous leishmaniasis in an emerging focus in North Morocco: new clinical forms. Ann Dermatol Venereol. 1999;126:419–22. (in French). 10434105

[35] Rhajaoui M, Nasereddin A, Fellah H, Azmi K, Amarir F, Al-Jawabreh A (2007). New clinicoepidemiologic profile of cutaneous leishmaniasis. Morocco Emerg Infect Dis.

[36] Assis RR, Ibraim IC, Noronha FS, Turco SJ, Soares RP. Glycoinositolphospholipids from *Leishmania braziliensis* and *L. infantum*: modulation of innate immune system and variations in carbohydrate structure. PLoS Negl Trop Dis. 2012;6:e1543. 10.1371/journal.pntd.0001543 PMC3289616 22389743

[37] McConville M, Schnur L, Jaffe C, Schneider P (1995). Structure of *Leishmania* lipophosphoglycan: inter-and intra-specific polymorphism in Old World species. Biochem J.

[38] Passero LF, Assis RR, da Silva TN, Nogueira PM, Macedo DH, Pessoa NL (2015). Differential modulation of macrophage response elicited by glycoinositolphospholipids and lipophosphoglycan from *Leishmania* (*Viannia*) *shawi*. Parasitol Int.

[39] Ghoshal A, Gerwig GJ, Kamerling JP, Mandal C. Sialic acids in different *Leishmania* sp., its correlation with nitric oxide resistance and host responses. Glycobiology. 2010;20:553–66. 10.1093/glycob/cwp207 20085901

[40] Moreira D, Rodrigues V, Abengozar M, Rivas L, Rial E, Laforge M (2015). *Leishmania infantum* modulates host macrophage mitochondrial metabolism by hijacking the SIRT1-AMPK axis. PLoS Pathog.

[41] Resende M, Moreira D, Augusto J, Cunha J, Neves B, Cruz MT, et al. *Leishmania*-infected MHC class IIhigh dendritic cells polarize CD4+ T cells toward a nonprotective T-bet+ IFN-γ+ IL-10+ phenotype. J Immunol. 2013;191:262–73. 10.4049/jimmunol.1203518 23729437

